# Thioredoxin-1: A Promising Target for the Treatment of Allergic Diseases

**DOI:** 10.3389/fimmu.2022.883116

**Published:** 2022-04-28

**Authors:** Jinquan Wang, Jiedong Zhou, Cuixue Wang, Atsushi Fukunaga, Shujing Li, Junji Yodoi, Hai Tian

**Affiliations:** ^1^ Department of Basic Medicine, Medical College, Shaoxing University, Shaoxing, China; ^2^ Division of Dermatology, Department of Internal Related, Kobe University Graduate School of Medicine, Kobe, Japan; ^3^ Laboratory of Infection and Prevention, Department of Biological Response, Institute for Virus Research, Kyoto University, Kyoto, Japan; ^4^ Department of Research and Development, Jiaozhimei Biotechnology (Shaoxing) Co., Ltd., Shaoxing, China

**Keywords:** allergic disease, anti-inflammatory, glucocorticoid resistance, migration inhibitory factor, thioredoxin-1

## Abstract

Thioredoxin-1 (Trx1) is an important regulator of cellular redox homeostasis that comprises a redox-active dithiol. Trx1 is induced in response to various stress conditions, such as oxidative damage, infection or inflammation, metabolic dysfunction, irradiation, and chemical exposure. It has shown excellent anti-inflammatory and immunomodulatory effects in the treatment of various human inflammatory disorders in animal models. This review focused on the protective roles and mechanisms of Trx1 in allergic diseases, such as allergic asthma, contact dermatitis, food allergies, allergic rhinitis, and drug allergies. Trx1 plays an important role in allergic diseases through processes, such as antioxidation, inhibiting macrophage migration inhibitory factor (MIF), regulating Th1/Th2 immune balance, modulating allergic inflammatory cells, and suppressing complement activation. The regulatory mechanism of Trx1 differs from that of glucocorticoids that regulates the inflammatory reactions associated with immune response suppression. Furthermore, Trx1 exerts a beneficial effect on glucocorticoid resistance of allergic inflammation by inhibiting the production and internalization of MIF. Our results suggest that Trx1 has the potential for future success in translational research.

## 1 Introduction

**Figure 1:**
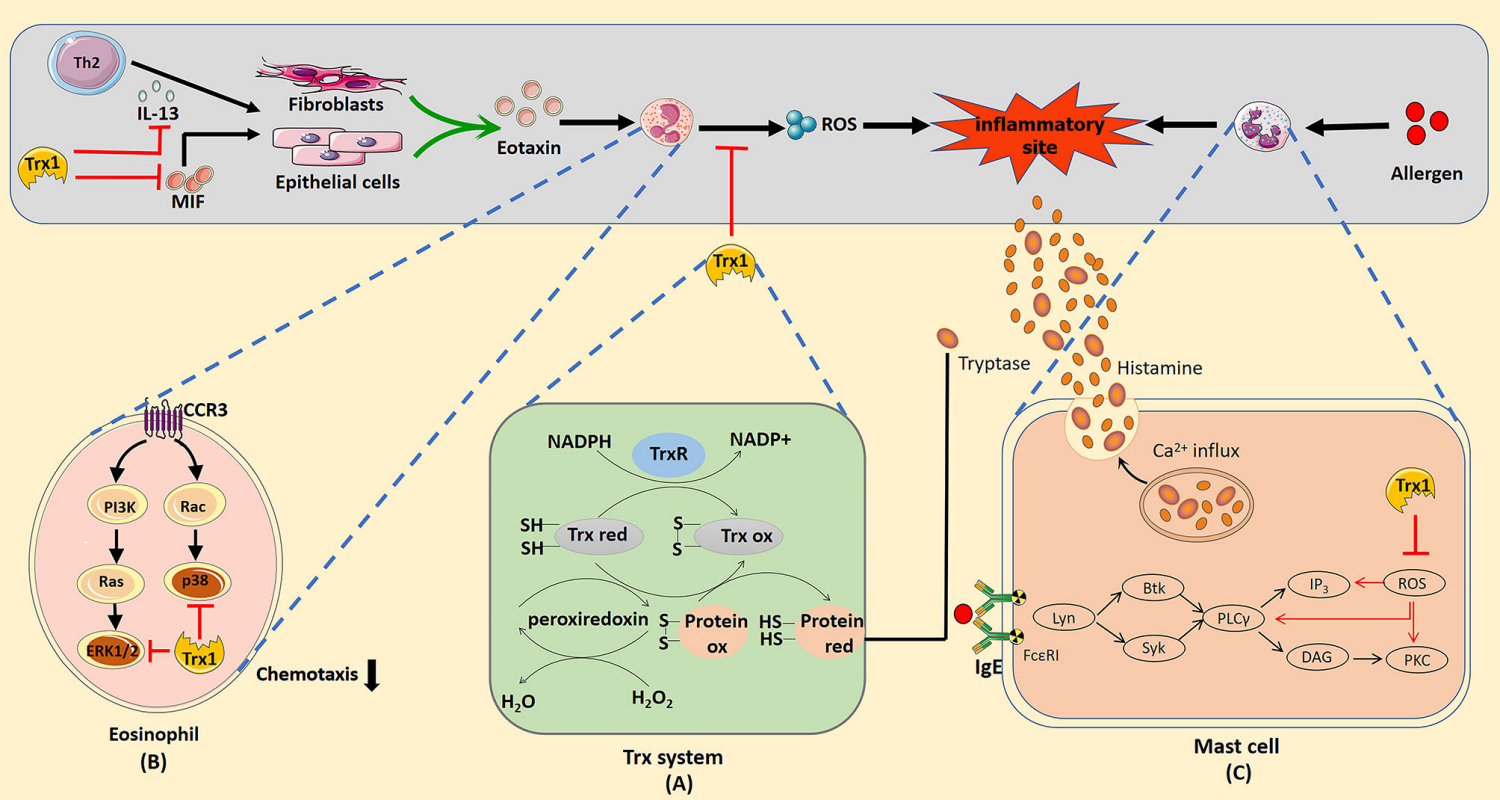
**(A)** Mechanism of redox regulation by the thioredoxin (Trx) system. The reduced form of Trx catalyzes the reduction of the disulphide bonds in the target protein. Oxidized thioredoxin is restored to its reduced state by the NADPH-dependent flavoenzyme thioredoxin reductase. **(B)** Trx1 inhibition of eosinophil activation and chemotaxis. Trx1 can eliminate reactive oxygen species (ROS) produced by eosinophils and directly inhibit the activation of the mitogen-activated protein kinase signal pathway when entering cells. Trx1 regulates Th2 response by inhibiting IL-13 production, which prevents IL-13 from stimulating epithelial cells or fibroblasts to produce eotaxin. In addition, Trx1 blocks the pro-inflammatory effect of the upstream chemokine macrophage migration inhibitory factor, which can directly induce the chemotaxis of eosinophils or promote the production of eotaxin by epithelial cells or fibroblasts to promote eosinophil recruitment. **(C)** Potential mechanisms of the effects of Trx1 on mast cell degranulation. Crosslinking of the allergen and IgE complex with FcϵRI activates the mast cell degranulation pathway, which then activates Lyn, Syk, Btk, and phospholipase Cγ (PLCγ). Activation of PLCγ eventually activates Ca^2+^ and protein kinase c (PKC), which contributes to degranulation. ROS induced in FcϵRI-stimulated mast cells activate mast cells by activating PLCγ, Ca^2+^ influx, and PKC. Accordingly, Trx1 prevents mast cell degranulation by scavenging ROS. The effective catalytic function of βII tryptase secreted by mast cells depends on the existence of normal disulphide bonds in molecules. The Trx1 system selectively reduces the number of disulphide bonds, which then reduces the catalytic activity of βII-tryptase.

Thioredoxin (Trx) is a ubiquitously expressed protein with a low molecular weight of 12 kDa. It is a part of the Trx system that includes NADPH and Trx reductase (TrxR) (1). Trx shows thiol–disulphide reductase activity that is influenced by a highly conserved active site (-Cys32-Gly-Pro-Cys35-) (2). The reduced form of Trx transfers its reducing equivalents to disulphides within the target molecule and catalyzes their reduction. TrxR uses NADPH in this process to reduce the active site disulphide in the Trx substrates to dithiol (3) (**Figure 1A**). Overall, the Trx system plays a critical role in regulating the cellular redox balance through the reversible thiol–disulphide exchange reaction.

Indeed, there are three distinct forms of human Trx, encoded by separate genes: cytosolic Trx (Trx1) and mitochondrial Trx (Trx2), and a spermatid-specific isoform of Trx (SpTrx/Trx3). Trx1 is a major isoform of Trx that is located in the cytoplasm but is also translocated to the nucleus. Trx1 can directly scavenge reactive oxygen species (ROS) and thereby protect against oxidative stress (4). It is also involved in various redox-dependent cellular processes, such as gene expression, signal transduction, and cell growth and apoptosis, and interacts with various target molecules (5). Under stress conditions, Trx1 is released into the extracellular space where it exerts a cytoprotective effect and shows cytokine-like activities (6). Trx2 is a mitochondrial redox protein sharing 35% sequence homology and similar catalytic properties with Trx1 *in vitro*, and possesses the Trx1 active-site but lacking additional structural cysteines (7). It plays a critical role for scavenging ROS to maintain a reducing status in the mitochondrial matrix (8).

Allergic diseases include immune-mediated disorders mainly characterized by a Th2 immune response phenotype. In patients with asthma, plasma Trx1 levels were significantly higher in the attack stage than those in the remission stage, and these levels of Trx1 substantially increased with the severity of the asthma attack (9). This implies that Trx1 can be a useful clinical parameter in asthma progression prediction.

The protective effects of Trx1 are associated with the pathogenesis of several human disorders, including metabolic syndromes and neurodegenerative, cardiovascular, and inflammatory diseases (10–13). In this review, we focused on recent studies on the underlying intercellular and intracellular mechanisms through which Trx1 regulates immune cells in response to allergic inflammatory diseases, such as allergic asthma, food and drug allergies, contact dermatitis, and allergic rhinitis (AR), as well as identifying the potential Trx-based therapeutic strategies for treating allergic diseases.

## 2 Therapeutic Effects of Trx1 on Allergic Diseases

### 2.1 Allergic Asthma

Airway inflammation in allergic asthma is a complex process, and Th2-type inflammation and excessive accumulation of eosinophils are the important features (14). At the site of airway inflammation, Th2 cells secrete large amounts of interleukin (IL)-3, -4, -5, -9 and -13 and recruit/activate eosinophils, mast cells, and basophils (15). IL-13 is crucial to the pathogenesis of asthma; overexpression of IL-13 significantly induces the occurrence of allergic asthma in a mouse model (16). Additionally, IL-13 induces not only the proliferation of goblet cells (the main effector cells for mucus production in the respiratory tract) but also subepithelial fibrosis which leads to airway remodeling (16, 17). Activated eosinophils migrate to the bronchial epithelium and release ROS and eosinophil granulocyte protein, resulting in airway hyper responsiveness (AHR) and epithelial damage that exacerbates the respiratory symptoms (18). The growth and survival signaling induced by ligand/receptor interactions in the airway smooth muscle cells are mediated through ROS (19). In addition, macrophage migration inhibitory factor (MIF), an important upstream regulator of airway inflammation, promotes eosinophil differentiation, survival, activation, and migration by binding to CD74 and CXCR4 on the surface of eosinophils (20). In the pathogenesis of allergic asthma, reduced expression of antioxidant genes is also observed, such as Nrf2. Clinical studies have shown that with the reduction of Nrf2 expression in the body, asthma becomes more serious (21, 22), and the application of Nrf2 agonists significantly relaxes the bronchi and improves the symptoms (23), suggesting that the occurrence of allergic asthma is not only related to Th2 inflammatory reaction but also has a close relationship with oxidative stress.

Clinical treatment of asthma mainly involves β2-receptor agonists, corticosteroids, and aminophylline. Although β2 agonists are currently the largest class of treatment agents for asthma, their use is controversial because of poor clinical reactions and possible life-threatening adverse events. For moderate and severe asthma, combination therapy with inhaled corticosteroids and long-acting β2-agonists is used; however, this combination cannot prevent, reverse, or treat the underlying causes of the disease. Moreover, this treatment requires continuous monitoring for side effects and resistance (24). For instance, aminophylline can often cause adverse reactions, such as palpitations, headache, and vomiting (25).

Trx1 is closely associated with asthma. Serum Trx1 levels in patients with acute asthma exacerbation are significantly high and there is a significant correlation between these levels and eosinophil cationic protein (9, 26). Exogenous Trx1 treatment can significantly improve AHR and airway inflammation in ovalbumin-sensitized mice (27). Similarly, in a mouse model of chronic asthma, the systemic use of Trx1 significantly inhibited airway remodeling, eosinophil infiltration, and AHR while reducing the expressions of eotaxin (an eosinophil chemokine), macrophage inflammatory protein-1 and IL-13 in the lungs; thus, Trx1 improves pathological changes in the airway to prevent remodeling and asthma development (28), in agreement with a recent report which indicated that Trx1 displayed pronounced protective effects on the manifestation of allergic airway inflammation (29). Trx1 also inhibits Th2 cytokine production by directly downregulating MIF production and indirectly inhibiting eosinophil chemotaxis. Notably, the realization of this process does not depend on the regulation of systemic Th1/Th2 immunity (30). The proliferation of goblet cells that secrete excessive mucus increases the morbidity and mortality of asthma patients; however, Trx1 prevents this proliferation or improves established goblet cell proliferation (31). Trx1 also regulates ARH and airway remodeling by directly reducing the production of intracellular ROS. Additionally, the clinical drug ephedrine may produce anti-asthma effects *in vivo* through inducing Trx1 production (32). The regulation of allergic asthma by Trx1 also involves the Trx1/Txnip system. Trx1 and Txnip are normally combined into a dimer, and the Trx1/Txnip dimer is separated in the presence of irritant factors such as inflammation. Txnip can activate such signaling pathways as ASK1, Bax, p38, and Caspase3, and induce apoptosis in the lung tissue (33). Moreover, Txnip can also activate inflammasomes to trigger inflammatory reactions (33), all of which aggravate the symptoms of allergic asthma. Trx1, on the other hand, inhibits these reactions, thereby maintaining the balance between Trx and Txnip. Overall, Trx1 may be useful for the treatment of asthma and may represent a therapeutic target for asthma control.

### 2.2 Allergic Rhinitis

The inflammatory process of AR is similar to that of asthma. Many Th2 cells infiltrate the nasal mucosa and release cytokines (e.g., IL-4, IL-5 and IL-13) that promote IgE production by plasma cells. A lot of medical treatment modalities used as a treatment of AR, such as antihistamines, steroids, montelukast, and immunotherapy. However, these therapeutic modalities can fail on some occasions (34).

Generally, large-scale production and release of inflammatory cells, including eosinophils and ROS and their metabolites, plays a vital role in the pathogenesis of allergic inflammatory airway diseases (35, 36). As an endogenous antioxidant protein, Trx1 has strong antioxidative stress effects. Thus, administration of exogenous Trx1 can inhibit AHR induced by specific allergens *via* the inhibition of eosinophil accumulation in the airway of mouse models with asthma (27, 28). Additionally, a recent study indicated that the concentration of Trx1 in nasal tissues is significantly decreased in patients with chronic rhinosinusitis with nasal polyps and a connection between elevated ROS levels and decreased levels of Trx1 has also been observed (37). Quercetin has been suggested as a dietary supplement for improving the clinical symptoms of allergic diseases, such as AR, but its precise mechanisms of action remain unclear. Nevertheless, Trx1 levels in the nasal mucosa significantly increase after oral administration of quercetin; moreover, the frequency of nasal allergy-like symptoms, such as sneezing and nasal rubbing, are significantly reduced (38). These changes provide insights into the possible mechanism underlying the favorable effects of quercetin on AR.

### 2.3 Food Allergies

Food allergies are caused by aberrant immune responses towards food antigens; these responses are skewed towards Th2 responses associated with IL-4, IL-5, and IL-13. Current treatments for IgE-mediated food allergies are largely confined to the avoidance of the suspected allergens, antihistamine treatments, and corticosteroid therapies with low efficacy and several side effects. Food allergen immunotherapy induces desensitization and promotes permanent immune tolerance to food allergens by gradually increasing exposure to the allergens (39); however, the incidence of adverse reactions is high and is a long-term treatment process (40).

Trx1 treatment has been effective against food allergies in previous studies. For example, the application of Trx1 significantly reduced allergic reactions in a wheat allergy dog model subjected to skin test. Therefore, Trx1 potentially reduces wheat sensitization by reducing the number of disulphide bonds in the major protein allergens of wheat (41). Similarly, Trx1 reduces the number of disulphide bonds in β-lactoglobulin, an allergen in bovine milk. The disulphide-reduced protein shows increased sensitivity to pepsin digestion and decreased hypersensitivity *in vivo* (42). Additionally, a Trx1-treated salt-soluble wheat allergen was shown to reduce IgE binding in children with asthma (43). Consistent with these results, active systemic and passive cutaneous anaphylaxis testing on guinea pigs showed that yeast extract rich in Trx1 significantly reduced egg mucin-induced anaphylaxis. It was hypothesized that the anti-allergic activity of Trx1 itself plays a role in these effects (44). Accordingly, Trx1-rich yeast extract can potentially be used to ferment foods, such as alcoholic beverages and bread. Recently, recombinant Trx1 rice has been shown to improve β-lactoglobulin digestion and decrease its allergenicity, thereby improving the feasibility and practicality of its large-scale application; a plant Trx system would be more cost-effective than those of *Escherichia coli* or animals (45).

### 2.4 Drug Allergies

Drug allergies (DAs) can be IgE- or non-IgE mediated. Some drugs, such as anesthetics, antibiotics, nonsteroidal anti-inflammatory drugs and codeine, are associated with a carrier protein through a prototype or its metabolite (46). Binding of cell-bound IgE molecules activates mast cells and releases various factors, such as histamines, leukotrienes, prostaglandins, and cytokines, which can cause extensive tissue damage. Trx1 is a stress-induced redox regulatory protein *in vivo*; thus, it inhibits histamine release by eliminating ROS in mast cells (47). The mechanisms of DAs may often be associated with non-IgE-mediated complement activation. Indeed, Trx1 is known to inhibit the activation of the complement cascade at different stages, e.g., suppressing C3 cleavage and C5 convertase activation (48, 49). The functions of Trx1 in mast cells and the complement system are described in section 3.5 “Suppression of Complement Activation”.

### 2.5 Contact Dermatitis

Contact dermatitis is a common inflammatory skin disorder that is usually characterized by alternating relief from and deterioration of symptoms, but it can be persistent at times (50). Contact dermatitis can be categorized as irritant contact dermatitis (ICD), a non-immunologically driven inflammatory reaction to an irritating substance, and allergic contact dermatitis, a type-IV delayed-type hypersensitivity reaction resulting from the activation of allergen-specific T cells, i.e., a second exposure to the allergen resulting in circulating memory T cells homing to the skin and eliciting an immunologic reaction that causes skin inflammation (51). Topical corticosteroid treatment is typically the first-choice treatment for contact dermatitis. However, corticosteroids are not suitable for long-term use because of multiple side effects, such as skin atrophy, telangiectasia, dermatoglyphics, and pigmentation. Oxidative stress is known to play a key role in contact dermatitis inflammation. In particular, ROS participate in dendritic cell activation (52). In addition to being an endogenous redox regulatory protein, Trx is an effective ROS scavenger (53). Because ROS regulate the function of dendritic cells that function in the sensitization phase of contact hypersensitivity, transgenic overexpression and systemic administration of exogenous Trx1 can suppress skin inflammation by inhibiting neutrophil recruitment during the elicitation phase, but not during the induction phase, in mice treated with 2,4-dinitrofluorobenzene (54). Transgenic overexpression of Trx1 and systemic administration of exogenous Trx1 can prevent the cutaneous inflammation caused by UV radiation through regulating the cellular redox status and ROS scavenging (55). We previously demonstrated that Trx1 ameliorates ICD by inhibiting epithelial production and releasing inflammatory cytokines and chemokines (56). Existing research suggests that Trx1 can be used to treat contact dermatitis; however, its exact therapeutic mechanism requires further clarification.

## 3 Trx1 Mechanisms

### 3.1 Eliminating ROS and Maintaining Redox Balance

Trx1 can directly remove ROS produced in inflamed tissues and help maintain redox balance. Mitsui et al. showed that Trx1 transgenic mice had strong resistance to oxidative stress and a longer life span compared with wild-type (WT) animals (57). Compared with the Trx1 system, the intracellular redox system has similar antioxidant mechanisms, such as the glutathione and peroxidase systems, which defend against oxidative stress. The Trx1 and glutathione systems act as backup systems to provide electrons for each other, i.e., the two systems protect cells from oxidative damage synergistically (58, 59). In addition, Trx1 is required to provide electrons when peroxidase is used to reduce ROS in organisms (60). Thus, Trx1 plays a key role in the balance of multiple redox systems in the body, and it coordinates the normal operation and function of these systems. Moreover, Trx1 is also a downstream target molecule for the activation of many redox signals. For example, in the case of mitochondrial redox imbalance, Nrf2 signal that is important for redox is activated (61). Nrf2 signaling activates some specific targets, including Trx1 and glutathione (62).

In the allergic state, expression of Trx1 can be induced to reduce the damage caused by excessive ROS. Simultaneously, the Trx1 system restores and refolds oxidized and damaged proteins. Consequently, Trx1 likely plays an important protective role against allergic inflammation.

### 3.2 Inhibition of MIF

Human MIF, a member of the Trx1 family of proteins that displays thiol reductase activity, was first cloned from T cells in 1989 (63). It shows inhibitory properties against the migration of macrophages and plays an essential role in cellular immunity, particularly in delayed-type hypersensitivity (64). MIF is largely considered a pleiotropic inflammatory medium with a wide range of immunoregulatory and pro-inflammatory activities, including the induction of inflammatory cytokines, regulation of macrophage and lymphocyte proliferation, and functions similar to those of chemokines (64, 65). Furthermore, MIF is directly involved in eosinophil differentiation, survival, activation, and migration (20).

MIF shares the redox-active motif -Cys-Xxx-Xxx-Cys- with Trx1 (66). It has sulfhydryl reductase activity and direct redox reactions with Trx1 (67). Several preclinical studies using animal models have shown that Trx has beneficial MIF-related functions against various inflammatory diseases. For example, the serum MIF level of Trx1 transgenic mice was significantly lower than that of WT mice in a dextran sodium sulphate-induced colitis mouse model (68). In mice with systemic inflammatory reactions from smoking, MIF gene expression in the spleens of Trx1 transgenic mice was inhibited compared with the expression levels in control mice (69). Using a mouse model of asthma, Torii et al. determined that MIF production in the lungs of Trx1 transgenic mice was significantly reduced despite similar systemic Th2 responses and IgE concentrations, indicating that Trx1 can suppress airway inflammation by directly inhibiting MIF independent of systemic Th1/Th2 immune modulation (30).

*In vitro* studies have provided evidence on the strong anti-MIF effect of Trx1. For instance, the production of MIF in macrophages cultured with LPS and IFN-γ was significantly inhibited by Trx1 (68). MIF expression is also suppressed in Trx1-transfected cells (70), and topically applied exogenous Trx1 suppresses the expression of MIF in ICD skin tissues (56). Additionally, MIF can enter cells to induce a series of inflammatory reactions, and cell surface Trx1 is one of the target proteins for MIF internalization. Specifically, Trx1 on the cell surface binds to extracellular MIF with high affinity and blocks MIF internalization. Exogenous and intracellular Trx1 can also directly bind to MIF, thereby forming a complex that blocks MIF-induced inflammatory response (71).

### 3.3 Regulating the Th1/Th2 Immune Balance

**Figure 2:**
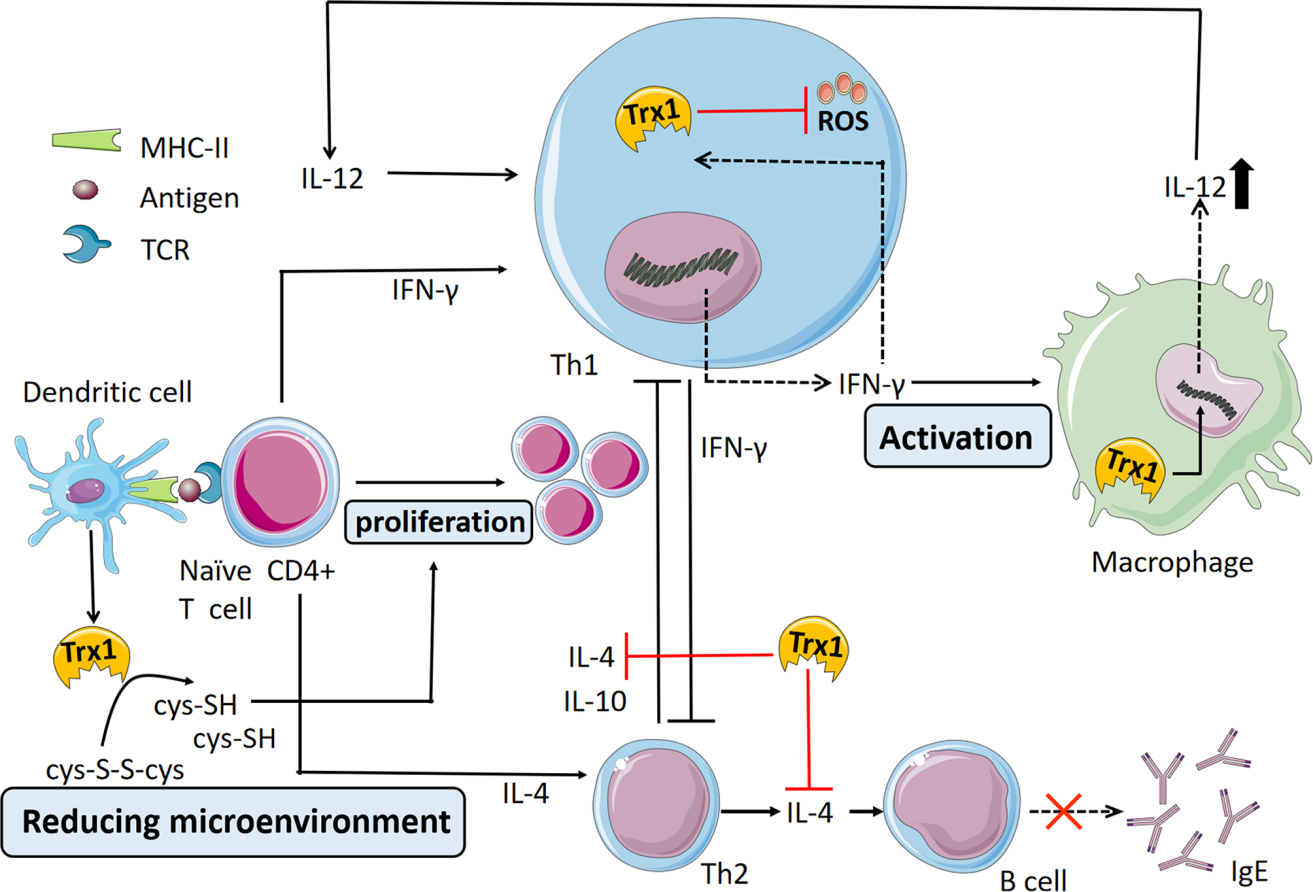
Thioredoxin-1 (Trx1) regulation of Th1/Th2 balance. Most extracellular cysteine (cys-SH) equivalents exist in the oxidized form of cystine (cys-S-S-cys). T cells cannot ingest cystine and rely on antigen-presenting cells (e.g., dendritic cells) to provide cysteine for them. Dendritic cells convert extracellular cystine into cysteine through Trx secretion, thereby promoting the proliferation of activated T cells. A positive feedback mechanism exists between Trx and IFN-γ wherein Trx1 induces the expression and release of IFN-γ in Th1 cells and the increased IFN-γ level increases Trx1 levels in turn. IFN-γ-activated intracellular Trx1 of macrophages increases Th1 cytokine IL-12 secretion by regulating the thiol redox state. Furthermore, Trx1 selectively inactivates the cytokine activity of IL-4 and inhibits the Th2 immune response.

In the cell microenvironment, proliferation and differentiation of Th1/Th2 cells are affected by various factors, such as cytokines, antigen properties, T cell receptor signal intensity, antigen-presenting cell types, and costimulatory molecules (72–74). In addition to these external factors, cell redox status is considered to play a role Th cell differentiation. T cells have limitations in terms of cystine uptake and require exogenous mercaptan for their activation to play a role in this process. During antigen presentation, after dendritic cells interact with T cells, the former generate and release Trx1, which reduces extracellular cystine to cysteine used by T cells; thus, the normal proliferative ability of T cells as well as an effective immune response are maintained (75, 76). Trx1 also controls the redox state of cell surface receptors, such as CD4 and CD30, and thereby affects the behavior of T cells (77, 78). When Th2 cytokine responses increase, Trx1 induces the expression of Th1-like cytokines, such as IL-1α, IL-1β, IL-1Ra, and IL-18, which in turn suppresses Th2-like cytokine expression (27). In recent studies, Trx1 has been confirmed as a specific target gene induced by the cytokine IFN-γ that directly drives the Th1 immune response (79, 80). Indeed, IFN-γ promotes Th1 differentiation and downregulates the Th2 response (81). Exogenous Trx1 can induce the expression and release of IFN-γ in Th1 cells, and the increased IFN-γ level in turn increases the Trx1 level. The intracellular Trx1 of IFN-γ-activated macrophages increases the secretion of the Th1 cytokine IL-12 by regulating the thiol redox state. Given the mutual induction and promotion of Trx1 and IFN-γ by immune cells during oxidative stress, a positive feedback mechanism could exist between Trx1 and IFN-γ as they participate in stimulating Th1 immunity (80). In addition, hTrx1 can bind to Dectin-1 and/or Dectin-2 on antigen presenting cells to secret IL-1β and IL-23, which influence Th2/Th17-polarizing milieu during the allergic sensitization in the skin (82). Recently, IL-4 has been identified as a new target of Trx1; specifically, its activity can be selectively suppressed by Trx1 (83) (**Figure 2**); thus, the production of IgE by B cells may also be effectively blocked. However, Trx1 does not directly affect the proliferation and differentiation of Th1/Th2 cells; instead, it suppresses inflammation by regulating the production and release of Th1/Th2 cytokines because lymphocytes isolated from Trx1-transgenic (Trx1-Tg) mice are similar to those from WT mice in terms of their ability to produce Th2 cytokines, such as IL-4, IL-5 and IL-13, once they leave an *in vivo* environment with high Trx1 (30). The recent report showed that regulatory T cells (Tregs) play an important role in maintaining immune tolerance to allergens by inhibiting the type 2 immune cells, inducing tolerogenic dendritic cells, regulatory B cells and IgG4-producing B cells in allergic disease (84). Increased Trx1 in Tregs enhances tolerance to oxidative stress (85). Thus, Trx1 may prevent the occurrence and progression of Th2-driven allergic inflammatory conditions by adjusting the Th1/Th2 balance. In response to various cytokines released by Th2 cells, such as IL-4, IL-5, IL-5 and IL-13, many inflammatory cells are activated, which in turn elicit an inflammatory response that leads to the clinical symptoms of allergic disease. The main effector cells are eosinophils, mast cells, and neutrophils in this complex immune response. A number of studies have shown that Trx1 directly modulate these cells through various mechanisms. Next, we focus on the role and the mechanisms of Trx1 on these inflammatory cells in allergic reactions.

### 3.4 Trx1 and Allergic Inflammatory Cells

#### 3.4.1 Eosinophils

Excessive proliferation and infiltration of eosinophils is generally considered a marker of allergic inflammation. Trx1 inhibits the migration and activation of eosinophils by regulating the extracellular Th1/Th2 balance, cellular signaling pathway, and molecules that interact with eosinophil-produced cytokines. In allergic asthma, Trx1 inhibits eosinophil accumulation by inducing Th1 cytokine production and suppressing Th2 cytokine production (27). Low expression of MIF in the airways of Trx1-Tg mice significantly inhibits eosinophil aggregation and mucus metaplasia (30). Additionally, MIF can directly induce the production of eotaxin to promote eosinophil chemotaxis (86); however, as described previously in the text, Trx1 can bind to MIF inside and outside the cells to block its internalization and pro-inflammatory activity. Eotaxin, an eosinophil chemotactic chemokine, is mediated by the C–C chemokine receptor type 3 (CCR3) on the surface of eosinophils (87). Eotaxin-stimulated eosinophils incubated with Trx1 significantly decreased the activation of eotaxin-stimulated ERK1/2 and p38MAPK pathways (88–90); however, Trx1 does not affect CCR3 expression in eosinophils. Thus, chemokine-induced eosinophil migration is apparently attenuated by regulating the downstream signaling of CCR3. In addition, intraperitoneal Trx1 injection significantly reduces the overproduction of MIP-1α and IL-13, which are closely related to eosinophil chemotaxis in the lungs (28). *In vitro* studies have confirmed that Trx1-overexpressing human bronchial epithelial cells can be protected from eosinophil-induced damage (91). Furthermore, Trx1 directly suppresses the production of ROS in eosinophils (92). Overall, Trx1 exerts anti-allergic effects by regulating eosinophil activation and migration (**Figure 1B**).

#### 3.4.2 Mast Cells

Mast cell activation plays an important role in various immediate allergic diseases. ROS functions in FcϵRI-mediated degranulation of mast cells (93, 94), and several ROS are generated during FcϵRI-mediated activation of mast cells. Thus, blocking the production of intracellular ROS can prevent the release of FcϵRI-mediated allergic mediators from rat mast cells (95). Son et al. stimulated mast cells from WT and Trx1-Tg mice with Ag, DNP-bovine serum albumin. The levels of histamine secreted by mast cells from Trx1-Tg mice were significantly reduced compared with that in WT mice, and the levels of intracellular ROS suggested that Trx1 inhibits mast cell degranulation by blocking ROS production (47). As the underlying mechanism, ROS mainly activates phospholipase Cγ (PLCγ), protein kinase C (PKC) and Ca2+ influx to cause medium release (94, 96), whereas Trx1 effectively inhibits PLCγ, PKC, and Ca2+ influx in the signal transmission of ROS-activated FcϵRI-dependent mast cells (**Figure 1C**).

βII-tryptase is one of the most abundant proteins stored and released in mast cells, and it participates in various acute and chronic allergic processes. It is commonly noted in patients with asthma and AR (97, 98). The redox activity of the allosteric disulphide bond (Cys220-Cys248 disulphide bond) in βII-tryptase plays an essential role in exerting enzyme activity, and Trx1 is a related βII-tryptase reducing agent *in vivo*; it can selectively reduce the disulphide bonds and potently reduce the catalytic activity of βII-tryptase in the reduced state (99) (**Figure 1C**).

#### 3.4.3 Neutrophils

Neutrophil recruitment is an important step in the pathogenesis of allergic sensitization and inflammation (100). Trx1 has an obvious inhibitory effect on the binding of neutrophils to vascular endothelial cells. Nakamura et al. found that Trx1 can inhibit the adhesion of neutrophils to endothelial cells in a mouse air sac chemotactic model (101). CD62L is an important adhesion molecule that is expressed and released by neutrophils, and it plays a key chemotactic role in neutrophil adherence to vascular endothelium and blood vessel penetration (102). Specifically, exogenous Trx1 acts directly on neutrophils, inhibiting the activation of the p38 mitogen-activated protein kinase (MAPK) signaling pathway, which causes the downregulation of CD62L in neutrophils, and ultimately reduces the adhesion of CD62L to endothelial cells. We previously explained its specific mechanism of action (103). Additionally, C32S/C35S mutant Trx1, showing a mutation at the redox function site, cannot inhibit the adhesion of neutrophils to human umbilical vein endothelial cells, indicating that the redox site of Trx1 is necessary for the inhibition of neutrophil adhesion (101). Moreover, in an LPS-induced bronchial inflammation rat model intravenously injected with 8 mg/kg of Trx1 every day, neutrophil infiltration into the bronchial and lung tissues had significantly reduced (104). Although adhesion molecules, such as ICAM-1, expressed by endothelial cells play important roles in neutrophil extravasation, Trx1 does not alter the expression of such adhesion molecules in these cells (101, 104). Therefore, Trx1 can inhibit the neutrophil recruitment by other chemokines, and it may play a unique role in neutrophil exudation of allergic inflammation.

### 3.5 Suppression of Complement Activation

Excessive complement activation has been implicated in the pathogenesis of allergic inflammatory disorders, such as IgE-independent DAs, and the increased production of the anaphylactic toxins C3a and C5a contributes to the activation of mast cells or basophils, vasodilation, and smooth muscle contraction. Transgenic overexpression of Trx1 *in vivo* or exogenous Trx1 injection can reduce choroidal neovascularization formation in laser-injured mouse models, which is closely associated with the complement activation of the Trx1 inhibition alternative pathway (48). Complement factor H, a multidomain and multifunctional protein, functions within the negative feedback that occurs during complement alternative pathway activation. It competes with factor B for C3b binding and accelerates the degradation of C3 convertase into its component (105). Trx1 inhibits C3 cleavage into C3a and C3b in a dose-dependent manner and prevents the deposition of C3b, and it inhibits the activation of C3b and reduces the generation of C3 convertase by binding to complement factor H; thus, it enhances the inhibition of C3 cleavage by complement factor H (48).

**Figure 3:**
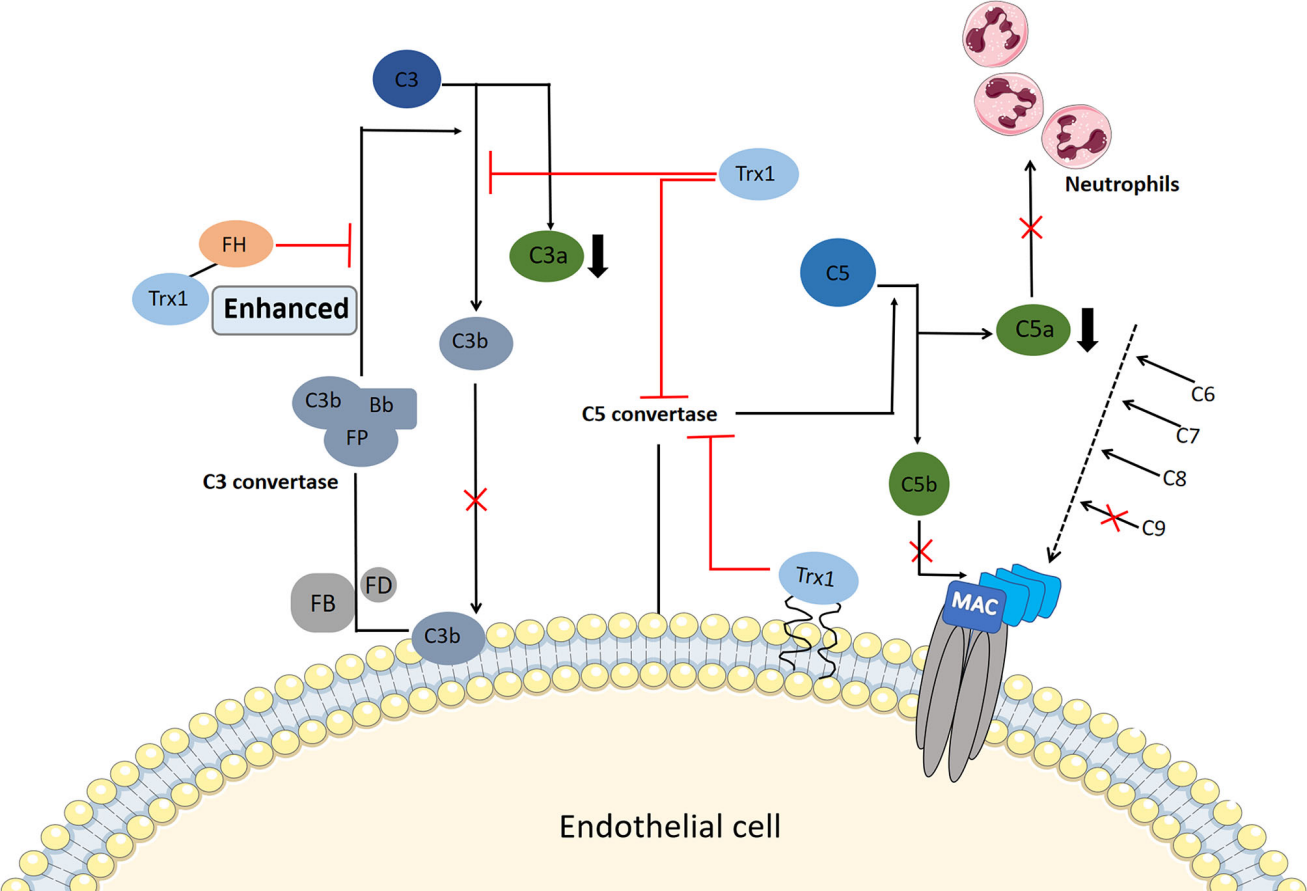
Potential mechanism of thioredoxin-1 (Trx1) inhibition of complement activation. Serum Trx1 inhibits C3 cleavage in the alternative pathway alone and enhances factor H (FH)-induced inhibition of C3 cleavage by combining with FH, which reduces C3a levels and C3b deposition. In contrast, Trx1 on the surface of endothelial cells or serum Trx1 blocks the production and deposition of C5b by inhibiting C5 convertase activity in the three complement terminal pathways; moreover, C9 deposition is inhibited. At the same time, Trx1 inhibits the production of anaphylaxis toxin C5a, which reduces the chemotaxis of neutrophils.

Moreover, Trx1 inhibits the activation of C5 convertase through its active site, thereby preventing the production of C5a and the formation of the membrane attack complex (49) (**Figure 3**). The deposition of C5b and C9 is also inhibited by Trx1 in a concentration-dependent manner in all three pathways during their early stages; however, Trx1 does not inhibit the deposition of non-allergic toxin C3b, which has a conditioning effect on bacteria and promotes phagocyte phagocytosis (106). C5a shows strong chemotactic activity in neutrophils and stimulates them to produce a large amount of oxygen free radicals, prostaglandins, and arachidonic acid. When Trx1 is intravenously injected into mice, complement-mediated neutrophil recruitment is significantly inhibited (48, 49). Therefore, blockage of complement activation by Trx1 may represent a therapeutic target for relieving IgE-independent allergic inflammation.

## 4 Trx1 Improves Glucocorticoid Resistance

Glucocorticoids (GCs), which stabilize mast cells to prevent degranulation and exert broad anti-inflammatory effects by binding to glucocorticoid receptors (GRs), are recognized as an effective first-line therapy for allergic diseases. Notably, GCs interfere with the division and proliferation of systemic lymphoid tissues under the action of antigens, affect the metabolism of lymphocytes, and induce lymphocyte apoptosis. Therefore, long-term administration of GCs attenuates host immunity to specific antigens and leads to the inhibition of the immune response to pathogenic microorganisms.

**Figure 4:**
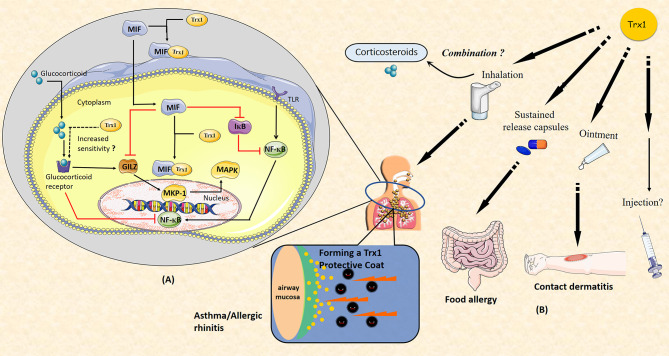
**(A)** Thioredoxin-1 (Trx1) improves glucocorticoid (GC) resistance through macrophage migration inhibitory factor (MIF). MIF impairs GC sensitivity *via* the inhibition of MAP kinase phosphatase-1 (MKP-1). MKP-1 is induced by GC to mediate GC inhibition of ERK, JNK, and p38MAPK activities as well as cytokine production. MIF inhibits GC-induced leucine zipper (GILZ) expression through unique effects on the expression of transcription factor and phosphorylation. MKP-1 and MAPK activation are regulated by MIF *via* GILZ. MIF also affects the NF-κB/IκB signal cascade. Trx1 may directly bind to GC receptor and enhance the response of cells to GCs. Both intracellular and extracellular Trx1 bind to MIF and form a heterodimer to prevent MIF entry into cells and MIF-induced GC resistance. **(B)** Potential clinical applications of Trx1 in allergic diseases. Administration of Trx1 suppresses the excessive allergic inflammatory response. Future clinical applications of Trx1 could include treatment of asthma or allergic rhinitis with a Trx1 inhaler, topical application for patients with contact dermatitis, and/or oral delivery for those with food allergies. It may also be promising to combine Trx1 with corticosteroids. Finally, Trx1 could potentially be administered as an intravenous injection.

In previous studies, we have shown that the anti-inflammatory and anti-allergic effects of Trx1 may inhibit host immunity, which is in contrast to the effects of corticosteroids (47, 54). Long-term use of GCs can cause GC resistance or insensitivity, which is a major obstacle in the treatment of allergic diseases. MIF can be induced by GCs and it enhances GC resistance (107); specifically, it impairs GC sensitivity *via* MAP kinase phosphatase-1 (MKP-1) inhibition (108). MKP-1 is an important MAPK signal inhibitor that is induced by GCs and mediates GC inhibition of ERK, JNK, and p38 MAPK activities as well as cytokine production induced by pro-inflammatory stimuli, such as LPS or IL-1 (109–111). MIF has been shown to downregulate GC-induced leucine zipper (GILZ) expression through a unique set of effects on transcription factor expression and phosphorylation. Notably, MIF-induced regulation of MKP-1 and MAPK activation is mediated through GILZ (112). Furthermore, MIF affects the NF-κB/IκB signal cascade, leading to accentuated inflammation and GC resistance (113). Trx1 can bind to GR and enhance the response of the cells to glucocorticoids (114). In addition, it can bind directly to MIF inside and outside the cell (71). Thus, Trx1 represents a potential intervention target between GC and MIF balance (**Figure 4A**).

## 5 Concluding Remarks

Trx1 induction is considered an effective compensatory protective mechanism through which damaged tissue proteins are reduced or repaired. Trx1 exerts anti-inflammatory effects on a wide variety of inflammatory disorders. In this review, we summarized the available data on Trx1 and highlighted a variety of mechanisms underlying its beneficial effects against allergic inflammation. Trx1 improved GC resistance, thereby acting as a promising therapeutic target both as a supplement to existing treatments for allergic diseases and for patients with hormone intolerance. In addition, it has been reported that increased levels of Trx1 in plasma or serum are correlated with the progression of diseases, especially allergic asthma. Thus, Trx1 may also serve as a potential diagnostic marker and be useful in prognostic assessments.

A variety of protein expression systems, including yeasts, lactobacillus, algae and plants cells, have been developed with anti-allergic and anti-inflammatory activities that are comparable to those found for purified recombinant human thioredoxin (rhTrx); thus, feasible sources for production of thioredoxin protein currently exist. In future translational research focused on Trx1, it will be essential to conduct human studies. Importantly, clinical trials are now ongoing in which rhTrx1 is being administered to patients with atopic dermatitis and trans-tracheal inhalation experiment with rhTrx1 are being performed; Trx1 is showing good efficacy with no major side effects (unpublished data). Finally, we suggest that Trx1 will be an important potential target for anti-allergic and anti-inflammatory drug development in the future (**Figure 4B**).

## Author Contributions

JW, JY, and HT were involved in the conception and writing of the manuscript, JZ, CW, AF, and SL contributed to literature searches and extensive discussions, and all authors agreed to publish the paper.

## Conflict of Interest

Author HT was employed by Jiaozhimei Biotechnology (Shaoxing) Co., Ltd.

The remaining authors declare that the research was conducted in the absence of any commercial or financial relationships that could be construed as a potential conflict of interest.

## Publisher’s Note

All claims expressed in this article are solely those of the authors and do not necessarily represent those of their affiliated organizations, or those of the publisher, the editors and the reviewers. Any product that may be evaluated in this article, or claim that may be made by its manufacturer, is not guaranteed or endorsed by the publisher.
